# Comparison of hydraulic, pneumatic and electric linear actuation systems

**DOI:** 10.1038/s41598-023-47602-x

**Published:** 2023-11-28

**Authors:** Jan Pustavrh, Marko Hočevar, Primož Podržaj, Ana Trajkovski, Franc Majdič

**Affiliations:** Faculty of Mechanical Engineering, University of Ljubljana, Aškerčeva 6, 1000 Ljubljana, Slovenia

**Keywords:** Engineering, Mechanical engineering

## Abstract

Different applications or industries use different systems for linear actuation, such as hydraulic, pneumatic or electric. Electric systems are becoming increasingly popular and are already replacing hydraulic systems in various applications. These are known to be potentially harmful to the environment, as large amounts of fluid can be released into the environment in the event of a pipe burst or other accident. This paper presents the results of a comparison between hydraulic, pneumatic and electric systems under variable conditions but with similar loads in all three systems. The common feature of all three systems is the input power, which was limited to 1.1 kW. There was no hydraulic accumulator in the hydraulic system and no pressure vessel in the pneumatic system, so no stored energy could influence the system behaviour or results. The main difference between the systems studied was the profile of displacement and power consumption. The most consistent response and lowest power consumption were obtained with the electric system, although both hydraulic and pneumatic systems can achieve consistent response with some modifications.

## Introduction

Hydraulic systems are commonly known to be potentially dangerous, as oil can leak into the environment in the event of a simple pipe burst or other accident or malfunction of the system^1^. In industry, hydraulic systems are increasingly being replaced by partial or complete electrification (aircraft, off-highway machinery, commercial vehicles, etc.) in order to reduce the impact on the environment, but on the other hand, hydraulic systems are still used in many industries^2–6^. Numerous studies are being carried out with the aim of replacing hydraulic fluids that are harmful to the environment with those that are compatible with nature and humans^1,7–11^. Pneumatic and electric systems are much more environmentally friendly as they have no direct impact on the environment. The latter systems are now being used more and more, and their capabilities are improving. A previous study^2,3^, compared and evaluated the characteristics of different servo motors using catalogues and non-public data, and another study^12^ compared three drive technologies (hydraulic, pneumatic and electric) for the same load positioning task. In study^13^, a simulation tool was used to compare the suitability of the choice between a hydraulic and an electric actuator on the Stewart platform. However, to our knowledge, comparable studies and experimental evaluations with hydraulic, pneumatic and electric linear actuators have not yet been carried out.

This paper presents a comparison of hydraulic, pneumatic and electric systems with linear actuators. Each system has its own characteristics and advantages that make it suitable for different applications. Hydraulic systems have become popular in industry because of their high force-torque ratio^3,14–16^. Actuators derive their energy from pressurised oil acting on the piston, which generates a force that sets the rod in motion^17^. They are usually executive components in hydraulic systems^18,19^. They are used in industry and manufacturing, as well as in heavy machinery and construction equipment (excavators, bulldozers, forklifts, telescopic ladders, etc.).

Pneumatic systems work on the same principle as hydraulic systems, with the difference that they use compressed air instead of oil^20^. Pneumatics are often used in automation technology, in applications that require a clean and dry environment. Recently, soft actuators have been increasingly used in automation systems to grip fragile products or semi-finished products^21^.

Electric actuators convert electrical energy (power) into mechanical energy or the rotary motion of a motor (servo or other) into linear motion via a screw and bearing system. They are used in a wide range of applications, from small domestic appliances to large industrial machinery. Their precision and control make them suitable for applications where precise and repeatable movement is required^20,22^.

Hydraulic^2,6,14,16,18,23–33^ and pneumatic^20,21,26,33–36^ systems are quite easy to compare because both systems convert the energy stored in the fluid into mechanical energy (force or torque) and the principle of operation is almost identical. The only difference is that the most common hydraulic systems usually use oil as the fluid, while pneumatic systems work with air. These two fluids have quite different properties (compressibility, density, etc.). It is much more difficult to compare an electric^2,9,14,20,22,37,38^ system with a hydraulic or pneumatic system, because the only commonality is the input power (power consumption) or the output force they produce. In this study, the limit was the input power (1.1 kW) without stored energy (hydraulic accumulator or pressure vessel).

## Experimental setup

**Figure 1:**
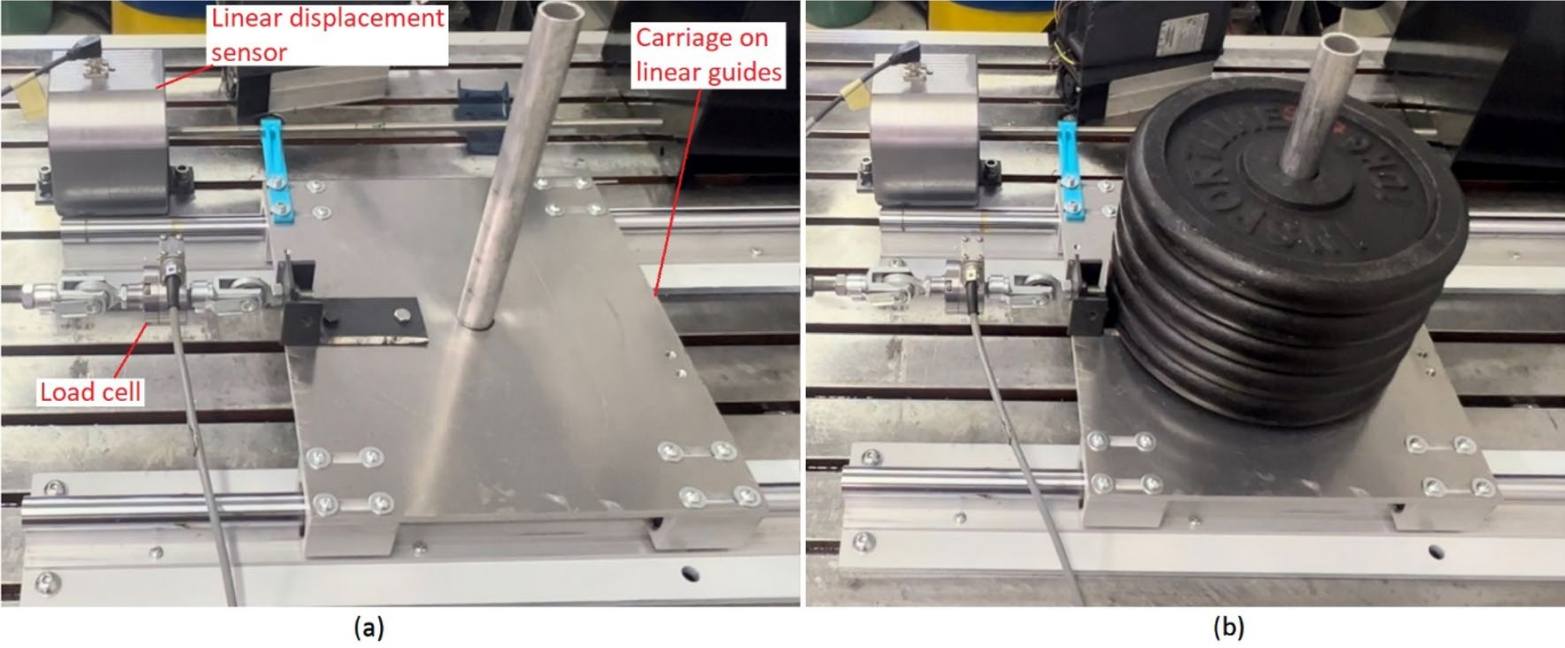
(**a**) Without load and (**b**) with additional load.

In order to be able to compare the hydraulic, pneumatic and electrical systems, the electrical input power was limited to 1.1 kW. The experimental part of each system is performed on a table with linear guides (Fig. 1a, b). It consists of the fixed part used for each actuator type (hydraulic, pneumatic, and electric), i.e. slides on linear guides on which we can place different masses (Fig. 1b) to vary the inertial mass (simulation of different loads), a linear displacement transducer (MAX48N-12V10EE0500, SICK), and a load cell (1-U2AD1/500 KG, HBM). The LabVIEW software environment with the National Instruments CompactRIO system was used for control and data acquisition (1000 Hz sampling rate). The individual system with actuator, control and measuring system is presented below.

The experiment is performed by applying a stepping function to the valve or servo motor of the electric actuator without a feedback loop. During the experiment, the piston rod is not allowed to reach its final or initial position (without bumping) when moving back, and the mass of the load is varied during the experiment (0 kg and 50 kg). In all three systems, the current consumption was measured with a 3-phase current sensor (AS050- SD -10V-3P, Loulensy Inc.).

In order to be able to compare all three systems and thus the response, it was necessary to define variable input data. For the hydraulic and pneumatic system this was the pressure at the pressure relief valve, for the electrical system, the maximum permissible acceleration was defined.

In the hydraulic system, the pressure relief valve was set to 50 bar, 100 bar and 200 bar; in the pneumatic system to 6 bar and 8 bar, and in the electrical system, the maximum permissible acceleration was set to 0.1 m/s^2^, 5 m/s^2^, 10 m/s^2^ and the maximum allowable value of the system to 15 m/s^2^.

### Hydraulic system

**Figure 2:**
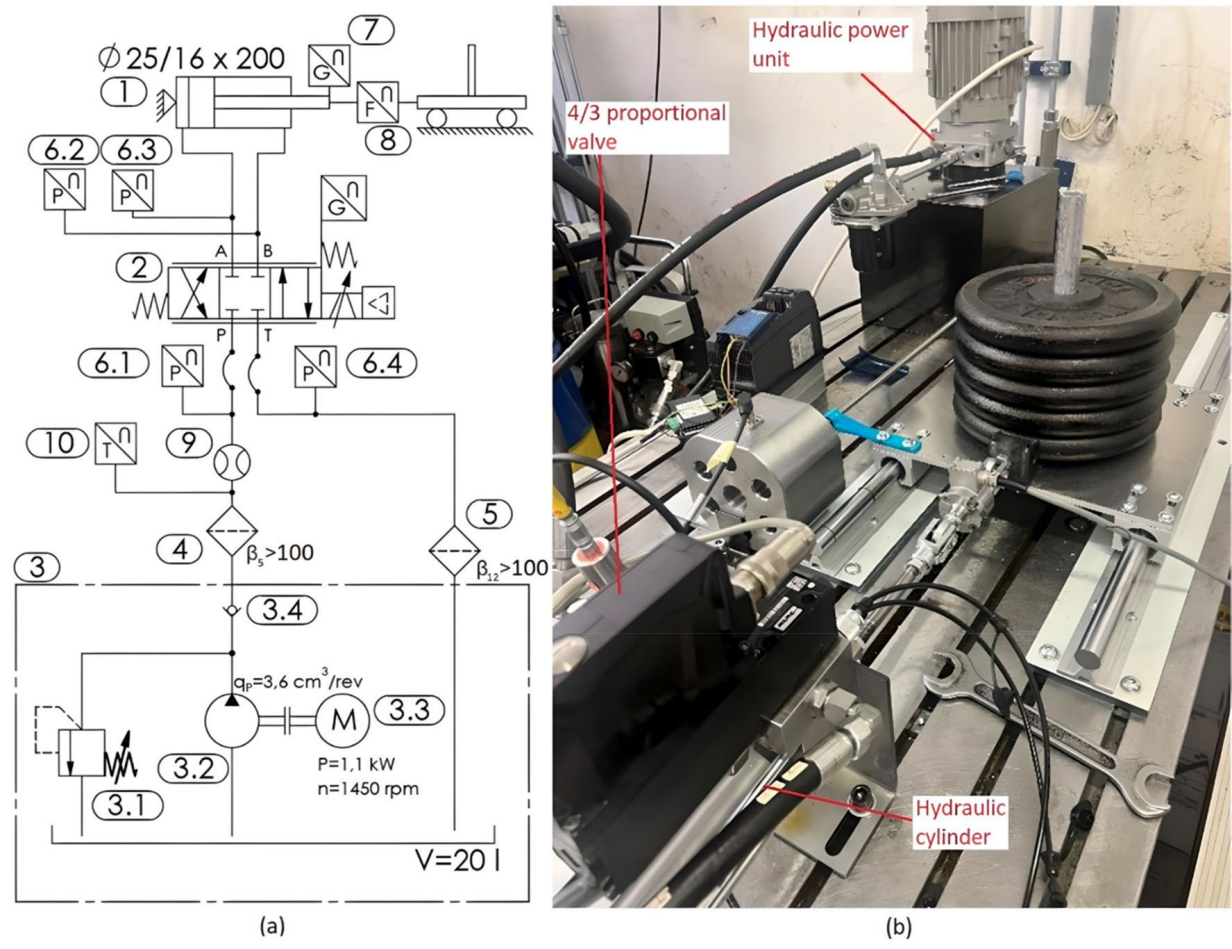
(**a**) Hydraulic system scheme and (**b**) actual hydraulic system.

The schematic of the hydraulic system is shown in Fig. 2a. The hydraulic system (Fig. 2b) consists of a double-acting hydraulic actuator (1) ϕ25/16 × 200, position 2 is a 4/3 proportional directional control valve (D1FPE01MC9NS0047, Parker), the hydraulic power unit (position 3) consists of a 1.1 kW electric motor (3.3) driving a gear pump (3.2) with a displacement of 3.6 cm^3^/rev (PGP502A0036CV1P1NE1B1E1B1, Parker), a pressure relief valve (3.1), a check valve (3.4), a high-pressure filter (4) and a return filter (5) to ensure cleanliness of the oil. Positions 6.1, 6.2, 6.3 and 6.4 are pressure sensors (SCP-400-74-02, Parker), position 7 is a linear position sensor (MAX48N-12V10EE0500, SICK), position 8 is a load cell (1 - U2AD1/500 KG, HBM), position 9 is a flow sensor (SCFT-060-02-02, Parker) and position 10 is a temperature sensor (SCT -190–04-02, Parker).

### Pneumatic system

**Figure 3:**
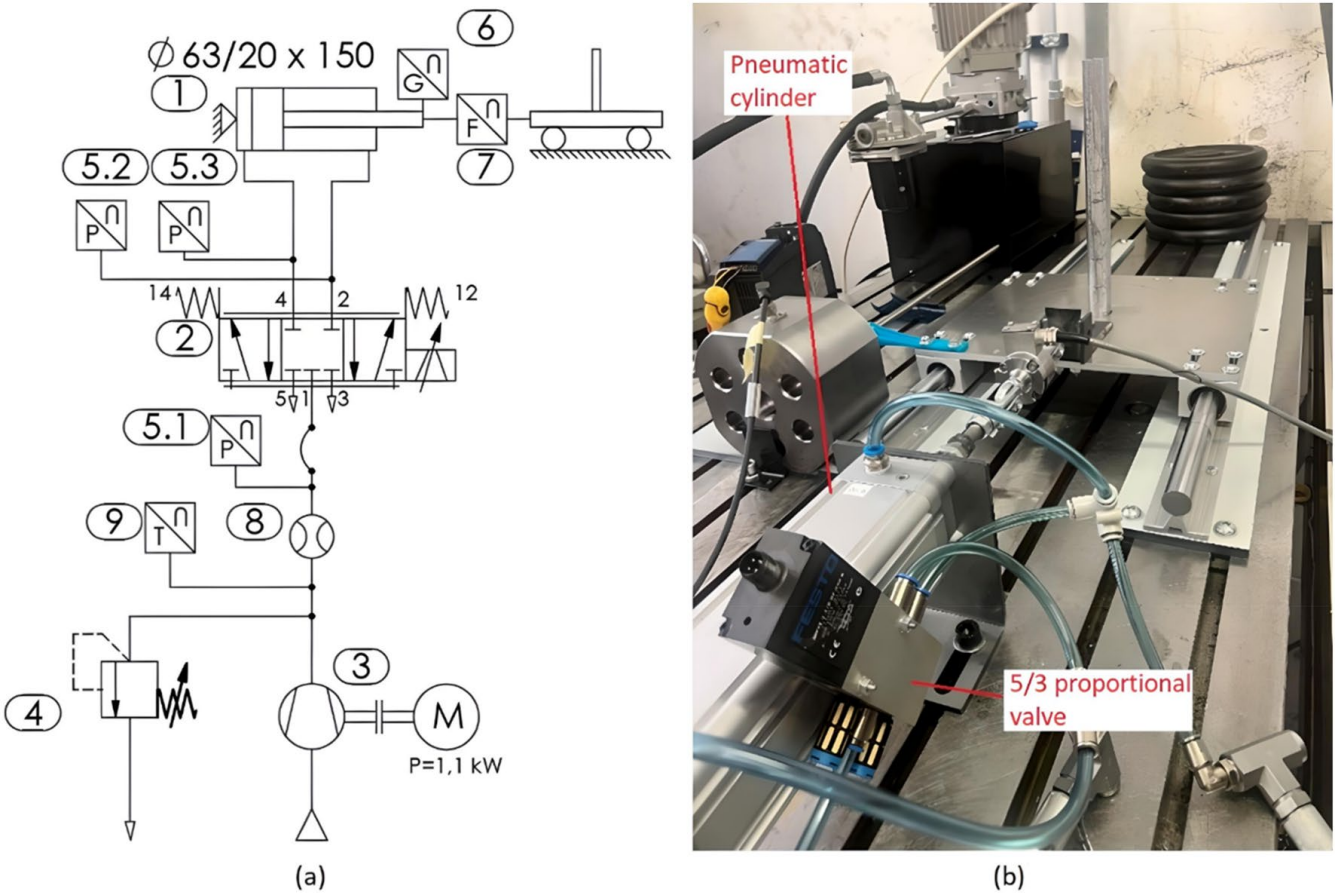
Pneumatic system scheme (**a**) and actual pneumatic system (**b**).

The pneumatic scheme is shown in Fig. 3. A double-acting actuator (1) ϕ63/20 × 150 (DNC-63-160-PPV-A, Festo) controlled by a 5/3 proportional directional control valve (2, MPYE-5-1/8-HF-010-B, Festo) was used. Position 3 is a 1.1 kW compressor for air supply without pressure vessel. Position 4 is a pressure relief valve, positions 5.1, 5.2 and 5.3 are pressure sensors (SCP-015-74-02, Parker), 6 is a linear position sensor (MAX48N-12V10EE0500, SICK), 7 is a load cell (1-U2AD1/500 KG, HBM), 8 is a flow sensor (PFM711-C8-F, SMC) and position 9 is a temperature sensor (SCT -190–04-02, Parker).

### Electrical system

**Figure 4:**
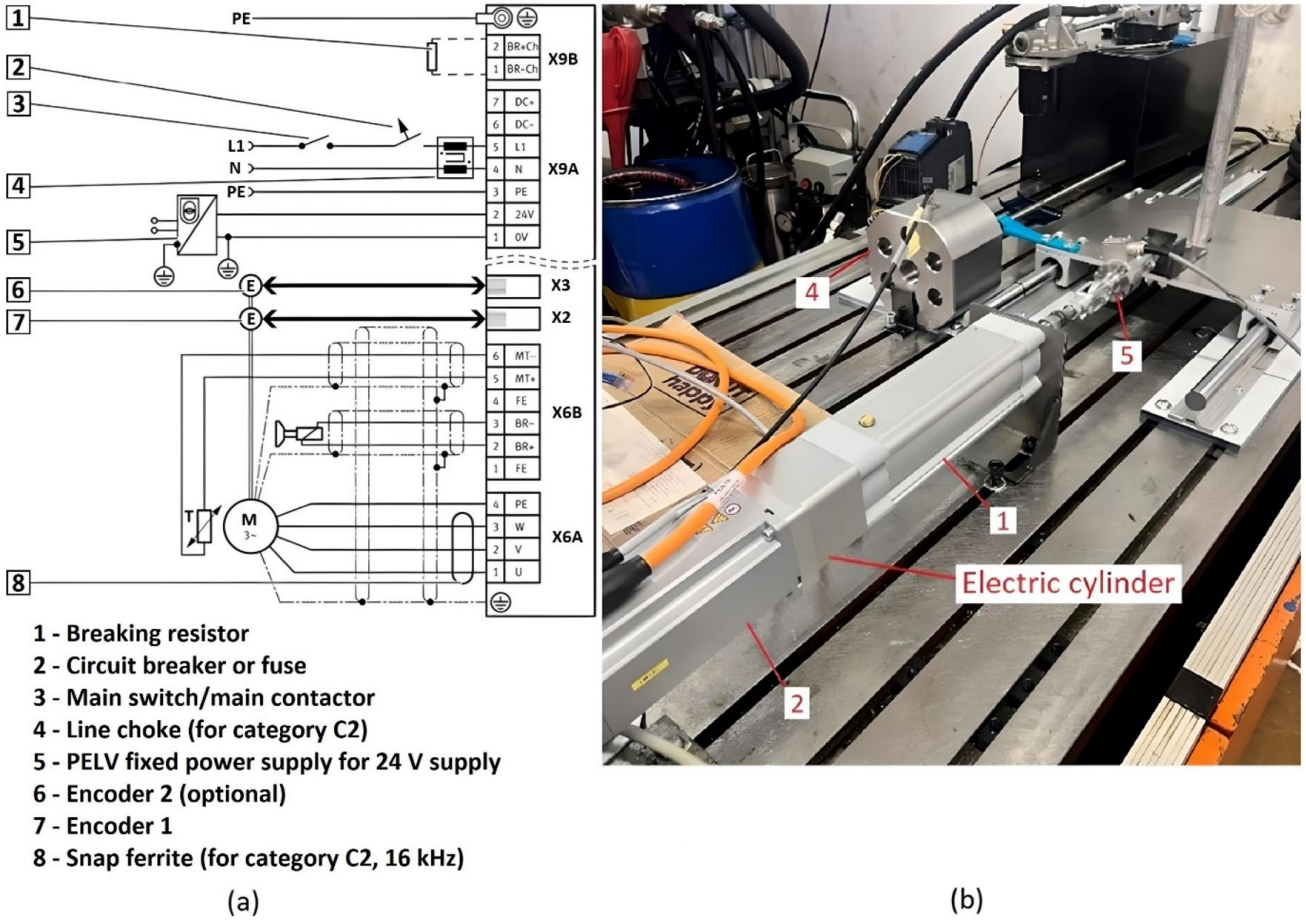
(**a**) Electric system scheme and (**b**) a photo of actual electric system^39^.

The electrical circuit diagram is shown in Fig. 4a. The electrical system (Fig. 4b) consists of an electric actuator (1, ESBF-BS -63-100-10P-S1-R3-F1, Festo). The servo motor (2, EMME-AS -80-S- HS-AMB, Festo) drives the lead screw, which converts the rotational movement into a translational movement. Position 3 is the servo drive controller (CMMT-AS -C2-3A- MP -S1, Festo), 4 is the position sensor (MAX48N-12V10EE0500, SICK) and 5 is the load cell (1-U2AD1/500KG, HBM). Unlike hydraulic or pneumatic systems, you only need cables for power transmission.

## Results

The results of the measurements on the individual systems are presented below.

### Hydraulic system

**Figure 5:**
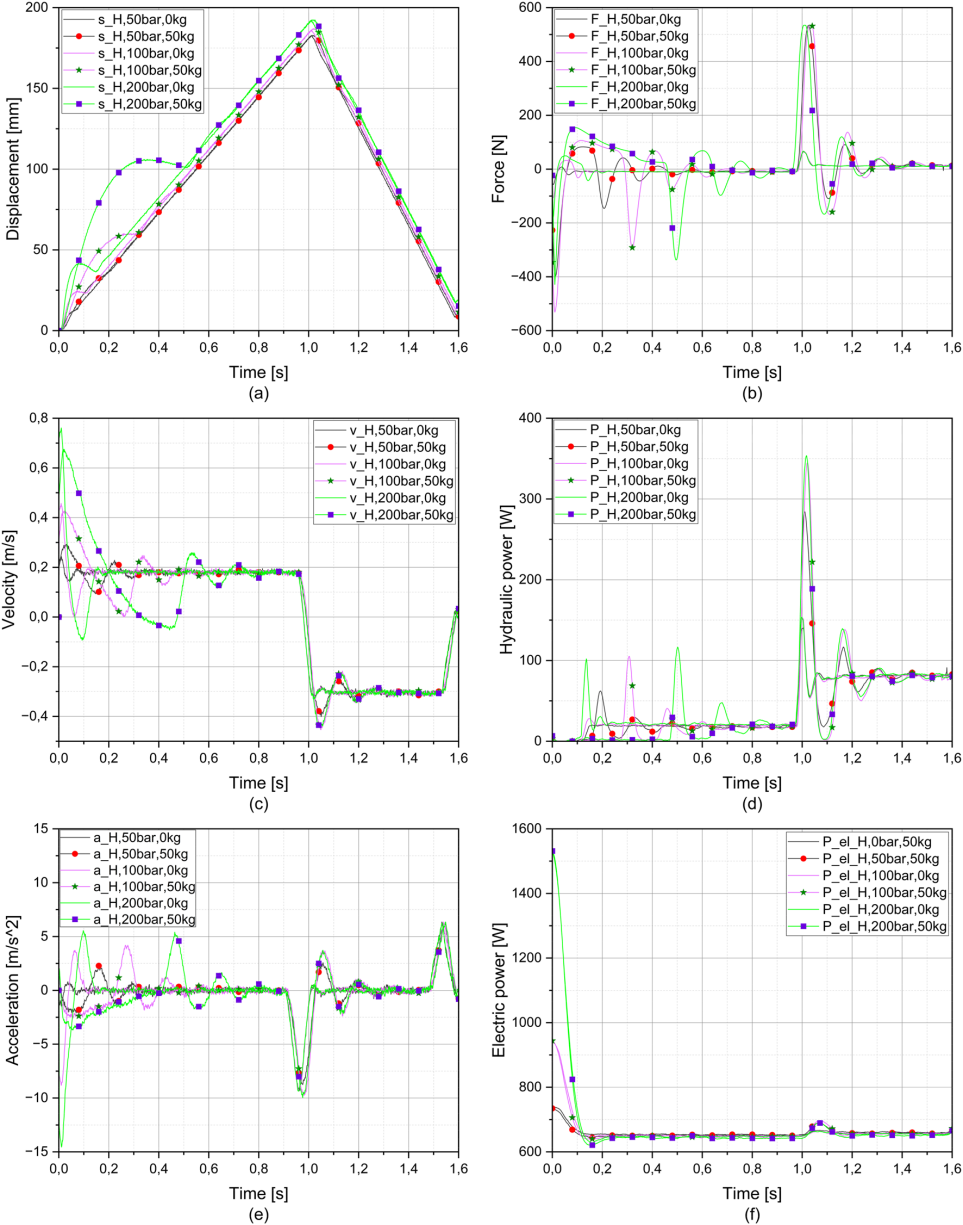
Charts of hydraulic measurements; (**a**) displacement, (**b**) force, (**c**) velocity, (**d**) hydraulic power, (**e**) acceleration and (**f**) electric power verses time.

Figure 5 shows the results of the measurements on the hydraulic system with a system pressure of 50 bar, 100 bar and 200 bar.

Since velocity is the first derivative and acceleration the second derivative of displacement, the deviations are clearly visible in both Fig. 5a, c and e. The maximum deviation from the expected (linear) value occurs at a system pressure of 200 bar in the unloaded state, with the highest velocity and acceleration and the lowest velocity and acceleration occurring at a system pressure of 50 bar in the unloaded state.

Figure 5c shows curves representing the measurements with and without load, and the measurements with load differ significantly. The oscillation frequency is significantly higher in the unloaded measurements than in the loaded measurements. The unloaded carriage settles after one period at constant velocity (0.2 m/s), while the period of oscillation of the loaded carriage decreases towards constant velocity (0.2 m/s) due to the influence of the inertial mass (acceleration and deceleration). At a system pressure of 200 bar, where this phenomenon is most pronounced, the period of oscillation is 0.15 s for the unloaded measurement. However, when the platform is loaded, three periods of oscillation occur, with the first period lasting 0.51 s, the second 0.17 s (about 35% of the first period) and the last period 0.16 s (about 32% of the first period). When the piston rod of the hydraulic cylinder moves towards the starting point, a similar behaviour as described above occurs. The loaded measurements show a lower oscillation frequency than the unloaded measurements, where the oscillation frequency decreases up to a constant velocity of 0.3 m/s. The backward velocity is higher due to the differential hydraulic cylinder (smaller surface area for the same flow). All this can also be seen in Fig. 5e (acceleration curves).

In Fig. 5b it can be seen that at the beginning of the movement of the piston rod there is a compressive force because the piston rod "pushes" the carriage away from itself, but soon the compressive force turns into a tensile force and the "belly" can be clearly seen in Fig. 5a. This means that the piston rod stops for a moment and the accelerated inertial mass wants to continue moving in the direction of the initial movement. For unloaded measurements, the compressive or tensile force is quite small, but increases as the system pressure increases. For measurements with load, the initial compressive force is much higher (20%) because the piston rod of the hydraulic cylinder has to push the load away from itself in addition to all the expected friction (seals, friction in the piping, etc.). After the initial compressive force, a higher tensile force occurs due to the greater inertia, but at the same time there is an oscillation between the tensile and compressive forces, with the frequency of the oscillation decreasing until the proportional valve is switched and the piston rod moves towards the starting point. During the measurements with the load, there is a sharp increase in the tensile force, which is due to inertia. At this point, the valve switches to the position where the piston rod starts to move back to the starting point, but the inertial mass continues to move in the other direction for a few moments. When approaching the starting point, the inertial mass "bumps" again, which is shown by the oscillation or transition between compressive and tensile force (the oscillation frequency decreases in the direction of constant friction).

The hydraulic power (Fig. 5f) depends on the flow rate and the pressure. When the valve is switched, the pressure drops to the value required to overcome pressure losses, friction, etc., and at this point flow enters the system. The oil begins to fill one chamber of the hydraulic cylinder and flows out of the other through the valve into the reservoir. At the beginning of the movement, the hydraulic power is "zero", which is mainly due to the energy or potential energy stored in the lines, even if the hydraulic accumulators have not been used. When the system pressure is increased, the hydraulic power also increases. In unloaded measurements, the hydraulic power was constant when the piston rod moved from the starting position (20 W), the increase only occurs at the beginning of the movement, but is very significant at a system pressure of 200 bar. Another immediate increase occurs when the valve is switched, when there is a small increase in pressure. When measuring with the load, the potential energy or pressure difference is so great that the hydraulic power is zero. This phenomenon is best observed at a system pressure of 200 bar, where the pressure difference is greatest. In the unloaded measurements, the power increase occurs after approx. 0.1 s, followed by a short increase and constant power; in the loaded measurements, the power increase only occurs after approx. 0.5 s. Similar to the unloaded measurements, there is a short power increase, after which the oscillation frequency drops to a constant power. As can be seen in Fig. 5f, when the system pressure is increased, the electric motor is loaded more before the valve is switched from the zero position, because the hydraulic pump generates a higher pressure. At a system pressure of 200 bar, the electrical power was about 1500 W before the piston rod started to move, while at 50 bar the electrical power was more than twice as much, i.e. about 740 W. After the valve was switched and the piston rod of the hydraulic cylinder started to move, the power dropped to about 650 W.

### Pneumatic system

**Figure 6:**
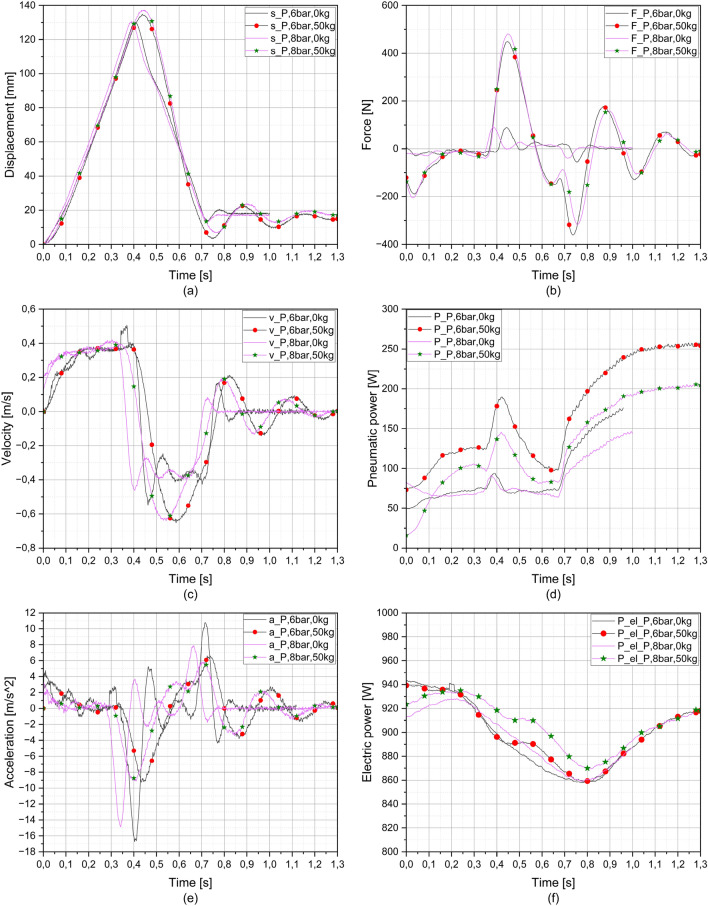
Charts of pneumatic measurements; (**a**) displacement, (**b**) force, (**c**) velocity, (**d**) hydraulic power, (**e**) acceleration and (**f**) electric power verses time.

Figure 6 shows the results of the measurement with a pneumatic system. The system pressure was set at 6 bar and 8 bar. With increasing system pressure, a similar displacement of the piston rod of the pneumatic cylinder was obtained. Figure 6a shows the influence of the compressibility of the air, especially for measurements with load.

The results of using the pneumatic system were surprising, as it was expected that the compressor would not be able to supply enough air because the reservoir (pressure vessel) was not used.

Figure 6c shows that the pneumatic system behaved better when the measurements were made with load. After switching the valve, the velocity increased to a constant value (0.35 m/s), while in the measurements without load, the velocity oscillated to the maximum value until the valve was switched. When the piston rod moved towards the starting point, an oscillation was detected in the unloaded measurements, which could be due to the compressibility of the air or to a lack of air. The lack of air can be seen in Fig. 6e, where the piston rod of the pneumatic cylinder accelerates and then cannot follow the movement due to the lack of air, resulting in oscillation. In the measurements with load, the velocity increased slowly (lower slope of the curve). Due to the slower acceleration at the beginning of the movement towards the end position, there was no sign of air shortage or oscillation, but the velocity was higher (0.6 m/s) than in the measurements without load, which is due to the inertial mass. When the valve was switched to the zero position, the effect of compressibility can be clearly seen in Fig. 6c and e, as the oscillation of the carriage with the inertial mass at the end can be seen.

In Fig. 6b, the compressive force is very low (− 20 N) in unloaded measurements where the piston rod has moved from its initial position, as the piston rod pushes the carriage away from itself. After switching the valve, there was an immediate increase in the tensile force, as the carriage continued to move in the direction of the initial movement for a few moments, after which the tensile force was very low again. In the measurements with load, the compressive force was ten times higher than in the unloaded measurements, but after the initial increase at the beginning, it decreased to very low values, very similar to those of the unloaded measurements. Due to the inertia of the mass, which continued to move in the direction of the initial position for a short time after the valve was switched (the piston rod began to move in the direction of the initial position), the tensile force increased sharply (480.5 N). The influence of the compressibility of the air is clearly noticeable in the measurements under load when the valve is switched to the zero position, as the movement of the inertial mass causes an oscillation between the compressive and tensile force (damped oscillation phenomenon, in which the oscillation frequency decreases towards zero with time).

Pneumatic power (Fig. 6d) is related to flow and pressure. In unloaded measurements, when the valve is switched, the pressure drops to the value required to overcome the friction in the guides, seals, etc. Therefore, the pneumatic power is quite constant (70 W), the increase only occurs when switching the valve due to a short pressure rise. With loaded measurements, the values of the pneumatic power are greater, because, in addition to the friction in guides, seals, etc., additional pressure is required to push or pull the carriage with weights.

The values of the electrical power (Fig. 6f) after the measurements with or without load do not differ, only with movement the power decreases. From this it can be concluded that the system is not overloaded.

### Electric system

**Figure 7:**
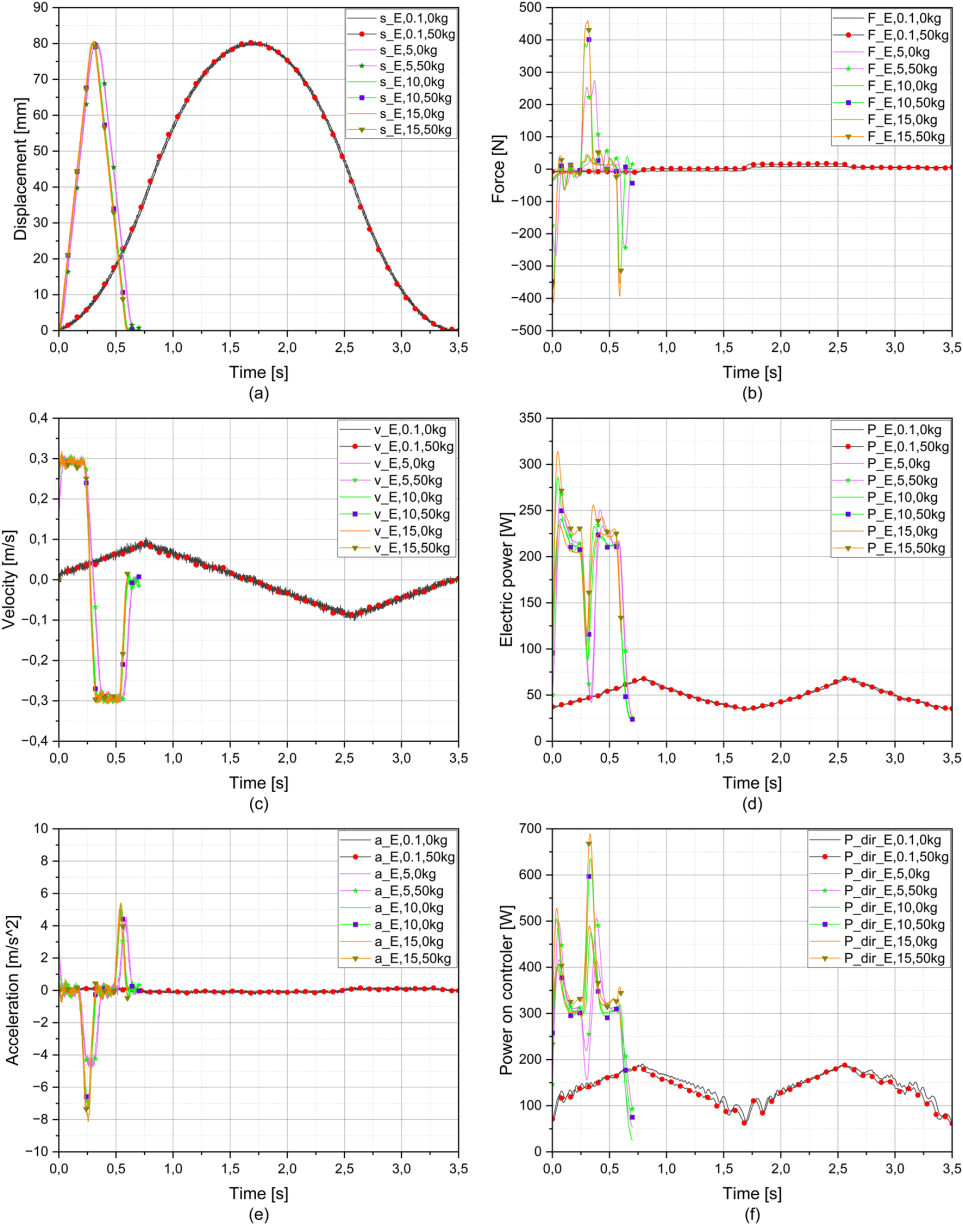
Charts of electric measurements; (**a**) displacement, (**b**) force, (**c**) velocity, (**d**) electric power, (**e**) acceleration and (**f**) electric power on controller time.

Figure 7 shows the results of the measurements on the electrical system.

At the limiting acceleration of 0.1 m/s^2^, a soft start can be seen in Fig. 7a. The piston rod of the electric cylinder begins to move slowly to the end position, while it reacts more jerkily when the limit acceleration is increased. Increasing the limit acceleration causes a similar displacement of the piston rod or the threaded spindle of the electric cylinder. Unlike the similar displacement, the permissible acceleration 0.1 m/s^2^ required 3.5 s to travel from the starting position to the end position and back without bumping. With the maximum permissible acceleration of 5 m/s^2^ and 10 m/s^2^, the extension and retraction was five times shorter and with the maximum permissible acceleration of 15 m/s^2^ almost six times shorter.

In addition to acceleration, the electrical system is also limited by velocity, which is about 0.3 m/s. This limitation can be seen in Fig. 7c. With a permissible acceleration of 0.1 m/s^2^, the maximum velocity was not reached because the piston rod or spindle accelerated so slowly and evenly that the maximum velocity was not reached. The maximum velocity was 0.1 m/s. Figure 7e shows that the measured values are within the configurable values, at the maximum permissible acceleration of 15 m/s^2^ the acceleration was not exceeded.

At a maximum permissible acceleration of 0.1 m/s^2^ as shown in Fig. 7a, c and 7e see also Fig. 7b), the acceleration was so smooth and slow that the force value is very low (from − 10 to 16 N). A different behaviour is visible when the maximum permissible acceleration is increased, especially for measurements under load. In unloaded measurements, a low compressive force (35 N) occurs initially, because the piston rod of the electric cylinder pushes the carriage out of itself, but at the same time friction in the guides, friction on the spindle in the electric cylinder etc. must be overcome. In the direction of the end position, the compressive force is very low. From the end position to the initial position, the piston rod pulls the carriage behind it, creating a tensile force. The initial tensile force is somewhat higher (35–45 N), but after the initial pull, the values drop to about 13 N. When the movement stops (deceleration), the carriage "bumps" against the piston rod, creating a compressive force. When measuring with the load, much greater compressive and tensile forces occur. The compressive force increases with the increase of the maximum permissible acceleration. Due to the inertia of the mass and the inability to follow the acceleration or movement of the inertial mass, there is an oscillation between the compressive and tensile forces (damped oscillation phenomenon). When the movement starts in the direction of the starting point, a high tensile force occurs due to the short movement of the inertial mass in the other direction. Here, too, an oscillation between the compressive and tensile forces occurs, because the inertial mass wants to move faster than the system allows due to the acceleration and "bumps" against the piston rod of the electric cylinder.

Figure 7d shows that the power increases with the increase of the maximum permissible acceleration, whereby the load has an additional influence on the power increase.

A similar effect can be observed with power measurements directly at the controller (Fig. 7f).

## Comparison of hydraulic, pneumatic and electric actuators

**Figure 8:**
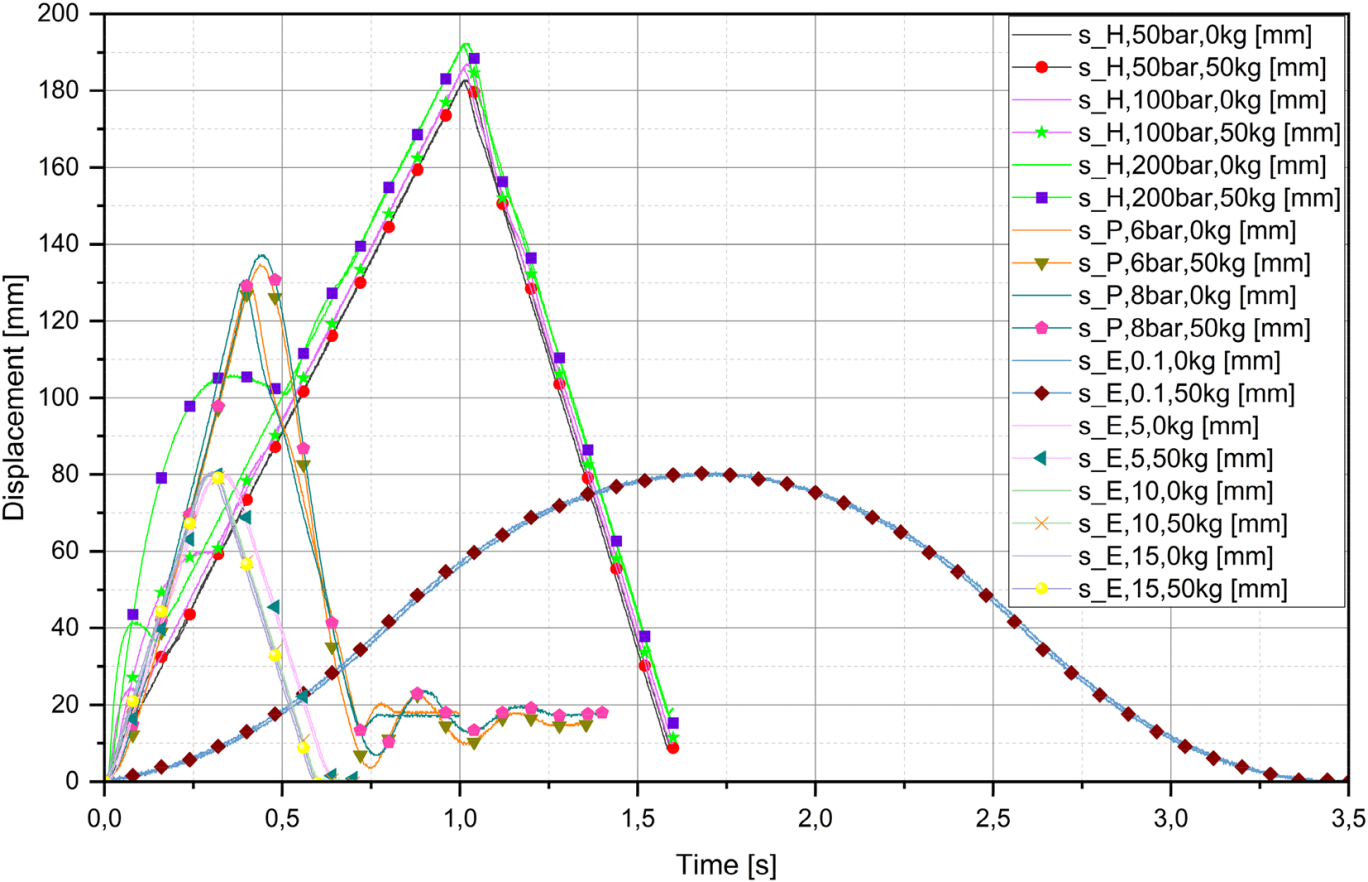
Displacement curves of all three systems.

The displacement curves of all three systems are shown in Fig. 8. Some curves deviate from the expected linear displacement ("belly" occurs). Since the first derivative of displacement is velocity and the second is acceleration, the same phenomenon can be is seen in Figs. 5, 6 and 7c and e. In the hydraulic system, this phenomenon is particularly evident due to the large pressure difference Δp. A pressure of 2 bar is required for the forward movement of the piston rod, and about 10 bar for the backward movement, measured with or without load. Increasing the system pressure (50 bar, 100 bar or 200 bar) leads to a significant increase in Δp. The increase in pressure difference affects the velocity and acceleration. When the valve is switched, all the potential energy stored in the pipe is released and the velocity immediately increases significantly (e.g. at a system pressure of 200 bar without load 0.75 m/s or 0.67 m/s for the measurements with load), but when the system settles, the velocity is constant (0.18 m/s). The fact that the velocities are higher at the beginning than in the pneumatic or electric system can be seen in Fig. 8, as the deviating curves have a steeper slope than the other curves. The increase in system pressure also affects the time it takes for the velocity to settle to the constant values (0.18 m/s). At 200 bar system pressure, it is 0.2 s without load and 0.9 s with load or just before the point where the valve is switched, i.e. at a time of 1 s. A similar oscillation occurs when the piston rod moves to the starting point, except that the pressure difference there is not as great as at the beginning of the movement (forward) when the valve was switched. The velocities are slightly higher (0.3 m/s) when the piston rod moves to the starting point, which is due to the smaller piston area and the same flow rate.

In the pneumatic system, the pressure drops from the system pressure (i.e. 6 bar or 8 bar) to about 1 bar at the beginning of the piston rod movement, and the values do not increase significantly as the piston rod moves towards the starting point. When the valve was switched at the beginning, the velocity or acceleration does not increase as much as in the hydraulic system because of the much smaller pressure difference. Figure 6c shows that the velocity increases to a constant value (0.37 m/s) when measured with load, while an oscillation is observed when measured without load. It is likely that the compressibility of the air causes the system to oscillate, as the frictional force in the guides increases during movement. A similar phenomenon occurs when the valve is switched or when the piston rod starts to move towards the starting point. Due to the smaller piston area (annular), higher velocities also occur in the pneumatic system. The velocity is higher in the measurement with load (0.65 m/s) than in the measurement without load (0.37 m/s), which is due to the influence of the mass inertia. In the measurements with load, when the valve is switched to the zero position at the end, the influence of the compressibility of the air can be seen, as a damped oscillation occurs. The velocity of the piston rod, to which the carriage with the additional mass is attached, decreases towards zero with time. Oil is also compressible (compressibility factor of oil 1.7 * 10^9^ Pa), but air is much more compressible (compressibility factor of air 1.4 * 10^6^ Pa)^40^.

The electrical system has no problem with the pressure difference or potential in the pipeline, because the system consumes as much energy (electricity) as it needs at that moment, whereas in the hydraulic or pneumatic system all the potential energy stored in the pipeline is released in the system when the valve is switched. During the measurements under load, some oscillations occurred, but the velocity quickly settled to a constant value of 0.3 m/s. In the hydraulic system, the energy peak is very noticeable (deviation from the expected linear movement of the hydraulic cylinder’s piston rod).

The velocity of each system can be determined from the slopes of the curves in Fig. 8; the steeper the slope, the higher the velocity. The curves of the hydraulic system have the greatest slope at the beginning, so that the velocity is the highest (0.76 m/s), and later the slope is the lowest, so that the constant velocity is the lowest (0.18 m/s). The pneumatic and electric systems have quite similar curve slopes, but the curves of the pneumatic system are slightly steeper, so that the velocity is slightly higher (0.37 m/s) than that of the electric system (0.3 m/s). Due to the change in piston area in the hydraulic and pneumatic systems, the return velocities are slightly higher. The slopes of the curves of the hydraulic and electric systems are quite similar, which is why the velocities are around 0.3 m/s. The pneumatic system has a slightly higher slope, and reaches a maximum velocity of 0.65 m/s during retraction.

Hydraulic and pneumatic systems are easier to compare because of similar characteristics (both convert the energy of a fluid into mechanical energy), while electrical systems convert electrical energy into mechanical energy. In all three systems, the influence of the inertial mass is evident in acceleration and deceleration (especially in the measurements under load). At the beginning, much higher compressive forces occur in the hydraulic system (− 530.9 N) than in the pneumatic (− 205 N) or electric system (− 413.1 N) due to the higher pressure differences. At the point where the valve has switched and the piston rods move backwards, but the inertial mass still wants to move in the other direction, very similar tensile forces occur (hydraulic 534.6 N, pneumatic 480.5 N and electric 459.5 N). The deviation in the hydraulic system may be due to the pressure shock, which causes a higher instantaneous acceleration and thus a higher tensile force. Oil and air are compressible, but when the piston rod of the pneumatic cylinder stops, there is much more oscillation than in the hydraulic system; in the electric system there is also oscillation, which can be caused by the factory-set control.

Figure 7d and f show that power increases from zero to a certain value. For example, the maximum power at the maximum permissible acceleration of 15 m/s^2^ was 233.6 W for the measurements without load and 313.9 W for the measurements with load. The power increases as the maximum permissible acceleration increases, and the system consumes more power for the measurements with load. In hydraulic (Fig. 5f) and pneumatic (Fig. 6f) systems, the power decreases or increases due to the continuous operation of the hydraulic power unit or compressor compared to the power required for continuous operation. In a hydraulic system, the power depends on the pressure setting of the pressure relief valve, as the electric motor is loaded more, similar to a pneumatic system, except that the difference between the pressures in the system is much smaller. The hydraulic system required 740.5 W of power at a system pressure of 50 bar before activation and 1529.9 W at 200 bar. After activation, the power dropped to about 650 W, indicating that a load of 50 kg is not a major obstacle for the hydraulic system, which is designed for much higher loads. For the pneumatic system, there are no significant differences in power at 6 bar (943.3 W) and 8 bar (923.3 W). When moving the piston rod forward and backward, the power did not drop as much as with the hydraulic system, indicating that increasing the load would overload the system.

Today, weight plays an important role in the selection of components. Hydraulic systems are considered robust and compact, but their biggest disadvantage is still the weight of the individual components. The total weight of a hydraulic system (described in the chapter Hydraulic system) is 52,029.3 g (100%), of a pneumatic system (described in the chapter Pneumatic system) 7946.5 g (15.3%) and of an electrical system (described in the chapter Electrical system) 11,636.9 g (22.3%).

**Figure 9:**
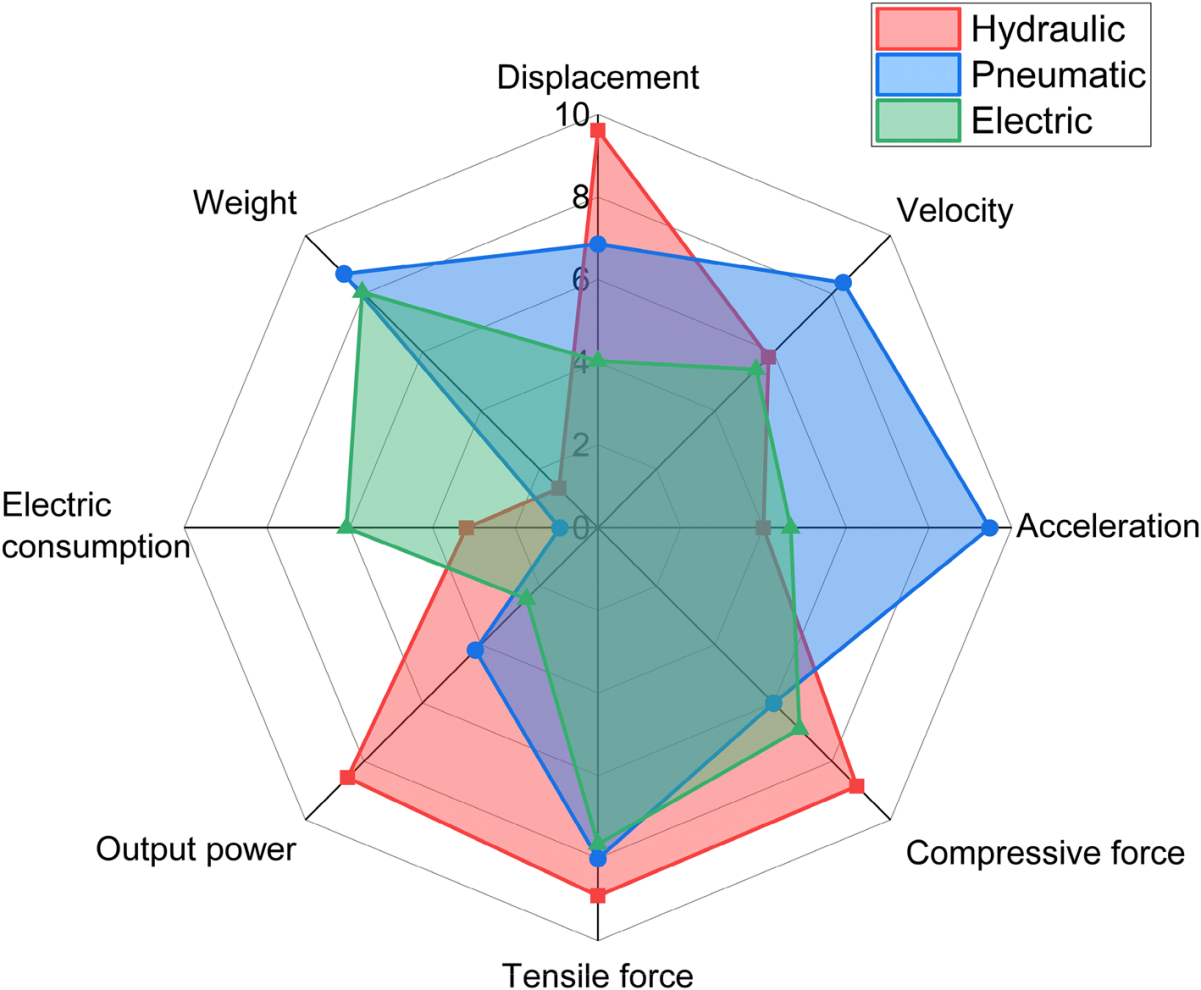
Ratings of the individual categories used to compare the three actuation systems.

Figure 9 shows the comparison of the eight most important parameters between hydraulic, pneumatic and electric systems. The radar chart shows the ratings for each category (velocity, acceleration, force, etc.). The ratings are given from 0 to 10. The displacement rating is directly related to the actuators used (grade 0 = 0 mm, grade 10 = 200 mm), so the hydraulic actuator gets the best rating (9.61) and the electric actuator the worst (4.03). For the evaluation of the velocity (rate 0 = 0 m/s, rate 10 = 0.5 m/s), the average velocity for the execution of the working or backward movement is used. Here the pneumatic actuator received the highest rating (8.38) and the electric actuator the lowest (5.41). Similar to the evaluation of the velocity, the acceleration (rate 0 = 0 m/s^2^, rate 10 = 4 m/s^2^) was also evaluated according to the average acceleration value for the working or backward movement. Here too, the pneumatic actuator received the highest rate (9.47) and the hydraulic actuator the lowest (4.00). It can be assumed that the reason for the reversal is that the hydraulic system accelerated strongly at first and then could no longer keep up, resulting in a deceleration. The estimated force was divided into tensile and compressive force. For the compressive force, the hydraulic actuator (8.85) achieved the highest rate (rate 0 = 0 N, rate 10 = − 600 N) and the pneumatic actuator (6.01) the lowest. In terms of tensile force (rate 0 = 0 N, rate 10 = 600 N), the hydraulic actuator again achieved the highest rate (8.91) and the electric actuator the lowest (7.66). The opposite is the case when evaluating the power output and power consumption. The lower the rate, the higher the power output or power consumption. The average value was calculated for the power output. The highest rate (rate 0 = 300 W, rate 10 = 0 W) was achieved with the hydraulic actuator (8.56) and the lowest with the electric actuator (2.44). The average value was also determined for the power consumption. The pneumatic system consumed the most energy and was therefore rated 0.92 (rating 0 = 1000 W, rating 10 = 0 W), while the electric system consumed the least and was therefore rated 6.08. The last point of evaluation was weight. Here the hydraulic system received the lowest score (score 0 = 60,000 g, score 10 = 0 g) (1.33) and the pneumatic system the highest (8.68) as the lightest.

The diagram in Fig. 9 can be used to select the appropriate actuator or system when certain constraints or requirements are present.

## Conclusion

During experimental studies, several advantages and disadvantages of the three systems compared were found: hydraulic, pneumatic and electric.

The highest velocity was achieved with the hydraulic system (0.76 m/s), which then stabilised at 0.18 m/s when the piston rod moved forward, and because of the smaller piston area, the velocity was slightly higher when it moved backwards (0.3 m/s). When the pneumatic piston rod moved forward, the maximum velocity was 0.37 m/s, and the backward velocity of the piston rod was higher (0.64 m/s). As with the hydraulic system, the piston area of the pneumatic cylinder on which the air presses was smaller, so the backward velocity was higher. The electric system was limited to a maximum velocity of 0.3 m/s, which was reached when the piston rod moved forward and backward.

The main obstacle to achieving higher velocities in the hydraulic system is flow, which is limited by power, pump shaft revolutions, system elasticity/pressure accumulation and system parameters. The existing system could be upgraded with a frequency inverter to achieve a higher shaft revolution of the electric motor (and thus the pumps) and thus a higher flow. Flow is also important in a pneumatic system. The compressor compressed as much air as the system needed, so there was no bottleneck as expected (twitching of the pneumatic piston rod). To achieve smooth and constant movement in a hydraulic or pneumatic system (affecting speed, acceleration, etc.), the system must have a feedback loop controller that controls the opening of the valve (hydraulic or pneumatic) and opens or closes it as needed. The installation of hydraulic accumulators helps to prevent sudden releases of high energy potential or pressure shocks.

The advantage of the electric system is that the movement is much smoother, and therefore the velocities and acceleration. The velocities are the same in the forward and backward movement of the piston rod of the electric cylinder because the piston area does not change as in hydraulic and pneumatic systems. In hydraulic and pneumatic systems, this problem could be solved by using double-acting cylinders with double piston rods (same piston area during forward and backward movement). This problem can also be solved by controlling the flow, although in highly dynamic systems it is easier to use double-acting cylinders with double piston rods. However, the disadvantage of the electric system is that to achieve higher velocities, the entire system must be upgraded (larger servo motor, cylinder, controller, higher power, etc.). In hydraulic or pneumatic systems, the velocity can be increased to a certain extent by upgrading the hydraulic power unit or the compressor. To achieve significantly higher velocities, the valves, seals and guide elements in the cylinder must be replaced.

Another major advantage of the electric system is power consumption, as the system only uses as much power as it needs at any given time. Hydraulic and pneumatic systems require more power consumption because of the constant work of the hydraulic power unit and the compressor. The movement and therefore the velocity and acceleration of the electric system are more constant because the servo motor turns the spindle of the electric cylinder with a constant torque in a closed loop, whereas in hydraulic and pneumatic systems there are various pressure losses, pressure shocks, fluctuations in system pressure, etc. The servo motor that turns the spindle or piston rod of the electric cylinder is activated when it receives a signal, while the hydraulic power unit or compressor must work before the valve receives a signal. This can be seen in Fig. 5f, where the power before the valve is activated is 1529.9 W and drops to about 650 W after the valve has been switched and the piston rod is moving. In Fig. 6f, there is no significant drop in power before (maximum 943.3 W) and after (minimum 859.2 W) the valve is activated. If the hydraulic power unit or the compressor were to switch on at the moment the valve receives the signal, these two systems would be very slow and would not respond. The hydraulic system could handle even higher loads, depending on the results of the measurements, because the power during the forward and backward movement of the piston rod is much lower than before the valve was activated. The hydraulic system is more robust and can be used in heavy—dirty industries.

A very big disadvantage of hydraulic systems is the large footprint the system requires and the noise it makes. The hydraulic system is heavy and takes up more space because a hydraulic power unit is needed in addition to the actuator, valve, hoses, etc. The pneumatic system weighs only 15.3% of the weight of the hydraulic system, but still requires additional space for the compressor. The electric system weighs 22.4% of the weight of the hydraulic system, but requires minimal floor space as no hydraulic power unit or compressor is needed, only a power socket.

## Data Availability

The data sets supporting the conclusions of this article are included in the article.

## References

[1] Schneider MP (2006). Plant-oil-based lubricants and hydraulic fluids. J. Sci. Food Agric..

[2] Sakama S, Tanaka Y, Kamimura A (2022). Characteristics of hydraulic and electric servo motors. Actuators.

[3] Tanaka, Y., Sakama, S., Nakano, K. & Kosodo, H. Comparative study on dynamic characteristics of hydraulic, pneumatic and electric motors. in *ASME/BATH 2013 Symposium on Fluid Power and Motion Control* (American Society of Mechanical Engineers, 2013). 10.1115/FPMC2013-4459.

[4] Qiao G (2018). A review of electromechanical actuators for more/all electric aircraft systems. Proc. Inst. Mech. Eng. Part C J. Mech. Eng. Sci..

[5] Sarlioglu B, Morris CT (2015). More electric aircraft: Review, challenges, and opportunities for commercial transport aircraft. IEEE Trans. Transp. Electrif..

[6] Padovani D, Fresia P, Rundo M, Altare G (2022). Downsizing the electric machines of energy-efficient electro-hydraulic drives for mobile hydraulics. J. Phys. Conf. Ser..

[7] Müller-Zermini B, Gaule G (2013). Environmental approach to hydraulic fluids. Lubr. Sci..

[8] Olszak A (2020). Application of plant oils as ecologically friendly hydraulic fluids. Appl. Sci..

[9] Redekar A, Deb D, Ozana S (2022). Functionality analysis of electric actuators in renewable energy systems—A review. Sensors.

[10] Lovrec D, Kalb R, Tič V (2023). Application areas of ionic hydraulic fluids. Chem. Eng. Technol..

[11] Lovrec D, Tič V (2022). The importance of the electrical properties of hydraulic fluids. Ind. Lubr. Tribol..

[12] Dransfield P (1990). Comparing the responses of electric, pneumatic, and hydraulic servodrives. IFAC Proc. Vol..

[13] Noskievic, P. & Walica, D. Comparison of the stewart platform linear actuator variants using simulation tools. in *2021 22nd International Carpathian Control Conference (ICCC)* 1–6 (IEEE, 2021). 10.1109/ICCC51557.2021.9454621.

[14] Wiens T, Deibert B (2020). A low-cost miniature electrohydrostatic actuator system. Actuators.

[15] Uzny S, Kutrowski Ł (2019). Strength analysis of a telescopic hydraulic cylinder elastically mounted on both ends. J. Appl. Math. Comput. Mech..

[16] Pawlus W, Choux M, Hansen MR (2016). Hydraulic vs. electric: A review of actuation systems in offshore drilling equipment. Model. Identif. Control A Nor. Res. Bull..

[17] Stawinski L, Skowronska J, Kosucki A (2021). Energy efficiency and limitations of the methods of controlling the hydraulic cylinder piston rod under various load conditions. Energies.

[18] Skowrońska J, Kosucki A, Stawiński Ł (2021). Overview of materials used for the basic elements of hydraulic actuators and sealing systems and their surfaces modification methods. Materials.

[19] Nicoletto G, Marin T (2011). Failure of a heavy-duty hydraulic cylinder and its fatigue re-design. Eng. Fail. Anal..

[20] Sotoodeh K (2019). Actuator selection and sizing for valves. SN Appl. Sci..

[21] Davis S (2018). Pneumatic actuators. Actuators.

[22] Berri PC, Dalla Vedova MDL, Maggiore P, Riva G (2020). Design and development of a planetary gearbox for electromechanical actuator test bench through additive manufacturing. Actuators.

[23] Beovič, A. *Hidravlika*. (Jana, 1998).

[24] Fassbender D, Zakharov V, Minav T (2021). Utilization of electric prime movers in hydraulic heavy-duty-mobile-machine implement systems. Autom. Constr..

[25] Akers A, Gassman M, Smith R (2006). Hydraulic Power System Analysis.

[26] Parr, A. *Hydraulics and Pneumatics, Second Edition: A Technician’s and Engineer’s Guide*. (Butterworth-Heinemann, 1998).

[27] Aron, D. Electric rod actuators vs. hydraulic cylinders: A comparison of the pros and cons of each technology. https://www.tolomatic.com/info-center/resource-details/electric-rod-actuators-vs-hydraulic-cylinders/ (2016).

[28] Valilou, S. *Nonlinear Model and Control of Electro-Hydraulic Servo Systems* (University of Bergamo, 2017).

[29] Šulc, B. & Jan, J. A. Non linear modelling and control of hydraulic actuators. *Acta Polytech.* **42**, (2002).

[30] Yu Y, Zhang J, Meng X, Wang D, Ma S (2022). Effect of piston texture at inclination and eccentricity work conditions on damping characteristics of a hydraulic shock absorber. Sci. Rep..

[31] Zhao P, Xie A, Zhu S, Kong L (2023). Pressure optimization for hydraulic-electric hybrid biped robot power unit based on genetic algorithm. Sci. Rep..

[32] Hagen D, Padovani D, Choux M (2019). A comparison study of a novel self-contained electro-hydraulic cylinder versus a conventional valve-controlled actuator—part 2: Energy efficiency. Actuators.

[33] Shmouty M, Saadany M, Shmouty A (2023). Hydraulic and pneumatic control in mechatronics systems (article review). Delta Univ. Sci. J..

[34] Ponomareva, E. *Hydraulic and Pneumatic Actuators and their Application Areas*. (2006).

[35] Carneiro Falcão J, Bravo Pinto J, de Almeida FG (2020). Accurate motion control of a pneumatic linear peristaltic actuator. Actuators.

[36] Qi H, Bone GM, Zhang Y (2019). Position control of pneumatic actuators using three-mode discrete-valued model predictive control. Actuators.

[37] Zhang P (2010). Sensors and actuators. Adv. Ind. Control Technol..

[38] Boldea I (2004). Linear electromagnetic actuators and their control: A review. EPE J..

[39] Festo SE & Co. KG. CMMT-AS-C2/4–3A... Servo drive. https://www.festo.com/tw/en/a/8143163/ (2022).

[40] Jablonská J (2014). Compressibility of the fluid. EPJ Web Conf..

